# Determinants of dietary diversity and the potential role of men in improving household nutrition in Tanzania

**DOI:** 10.1371/journal.pone.0189022

**Published:** 2017-12-12

**Authors:** Justus Ochieng, Victor Afari-Sefa, Philipo Joseph Lukumay, Thomas Dubois

**Affiliations:** 1 World Vegetable Center, Eastern and Southern Africa, Arusha, Tanzania; 2 World Vegetable Center, West and Central Africa–Coastal and Humid Regions, IITA-Benin Campus, Cotonou, Benin; 3 Department of Applied Economics and Statistics, University of Delaware, Newark, Delaware, United States of America; TNO, NETHERLANDS

## Abstract

Good nutrition is a prerequisite for a healthy and active life, especially for agriculture-dependent households. However, diets in most households in Tanzania lack diversity because the intake of meat, poultry, fish, and vegetables and fruits is low. This study estimates factors influencing dietary diversity of the household, children under five years, and women using primary survey data. It qualitatively assesses male dietary patterns and men’s potential role in improving the nutritional status of the entire household. The findings show that the most consumed foods within the household are cereals, vegetables, oils and fats, spices, condiments and beverages. Children (*d* = 0.4; *p*<0.05) and women (*d* = 0.5; *p*<0.01) in female-headed households have low dietary diversity compared to those in male-headed households. Women and children access less diverse diets since 46% and 26%, achieved minimum dietary diversity respectively. Production of vegetables (coef. 0.34; *p*<0.05) play an important role in improving the dietary diversity of women. Gender (coef. 0.05; *p*<0.10) and education of the household head (coef. 0.02; *p*<0.01), food preparation and nutrition training (coef. 0.10; *p*<0.05) are important factors influencing dietary diversity of the members of a household. Results suggest that there is a need to support community-based programs to provide information on food and the importance of vegetables, their preparation, consumption and utilization to address food and nutrition challenges. Men can contribute towards improving household nutrition security by reducing consumption of food away from the home, especially during periods of food shortages. We recommend the use of complementary quantitative research to determine the patterns and dynamics of men’s dietary diversity and compare it with that of other household members.

## Introduction

Malnutrition is a problem affecting all countries and one in every three persons worldwide[1]. Consequently, ending all forms of malnutrition and providing access to safe, sufficient and nutritious food for all people year-round by 2030 is one of the targets of the United Nation’s’ Sustainable Development Goals (SDGs). Nutritional deficiencies are responsible for lower adult work productivity, impaired physical and mental development, susceptibility to various diseases, premature deaths in children and poor pregnancy outcome in women[2, 3]. Despite the fact that great progress has been made in improving food and nutrition security over the past decades, undernutrition causes 45% of child deaths globally [4] and the prevalence remains high, especially in sub-Saharan Africa (SSA) and South Asia [5] with its estimated average adult cost on these continents being equivalent to 8–11% of the gross domestic product (GDP)[1].

Undernutrition persists in SSA, where limited dietary diversity is a major challenge and the cause of malnutrition in rural and urban poor households [6,7]. Savy et al., [8]found that dietary diversity adequately represents the overall dietary quality of women in Burkina Faso and is positively associated with their nutritional status. Most households rely heavily on carbohydrate-rich staple crops and consume few animal products, fruits or vegetables, partially leading to increasing numbers of population experiencing malnutrition[6,7]. Although undernutrition affects both the urban and rural poor, those residing in rural areas face additional challenges such as social isolation, intermittent drought, limited participation of women in major economic activities, limited market access, poor rural health services [9], poor roads and over reliance on rain fed agriculture.

In Tanzania, undernutrition affects infants, children under five and women of child bearing age[10]. Currently, stunting (short of their age) affects about 34% of children under five in Tanzania, 5% are wasted (thin for their weight) and 14% are underweight (thin for their age) while about 5.5% of women aged 15–49 years are underweight[10]. Additionally, the burden of chronic undernutrition in Tanzania ranks third in SSA, after Ethiopia and the Democratic Republic of Congo[1]. For children, diversified diet is important because they need energy and nutrient rich foods for growth and a healthy life.

Research shows that undernutrition is particularly high among low income Tanzanian households, mainly because they consume carbohydrate-rich staple-based diets low in minerals and vitamins[11]. Consumption of staple foods provide more energy to households, but they are unable to adequately improve nutritional outcomes if not consumed together with micronutrient-rich foods such as beef, fish, poultry, fruits and vegetables. Besides, provision of energy-rich supplements to women during pregnancy leads to increased birth weight [12] but availability and affordability of these supplements is quite a challenge in rural areas of Tanzania. Households should not only consume adequate food quantities, but also safe and diversified foods. Most households in Tanzania depend on agriculture (more than 80% of the population) and can produce more fruits and vegetables for consumption to increase dietary diversity[13,14]. Vegetables, particularly the traditional types such as amaranths, African eggplant (*Solanum aethiopicum*), African nightshade (*S*. *nigrum*), okra (*Abelmoschus esculentus*), sweet potato leaves (*Ipomoea batatas*), pumpkin leaves (*Cucurbita maxima)* and jute mallow (*Corchorus olitorius*) are important sources of micronutrients, fiber, vitamins and minerals. Starchy staples provide more than 70% of the calorie intake of rural households in Tanzania [15] and 40% of the calories come from maize [16] indicating that important micronutrient rich foods such as meat, fish, dairy, eggs, fruits and vegetables are not being consumed in sufficient amounts by the households [10].

Dodoma and Mbeya are among the regions with the highest prevalence and number of stunted children in Tanzania. Overall about 45.2% of the children under 5 years in Dodoma and 36% in Mbeya region are stunted[10]. Yet the dietary diversity of rural households, children under five and women (15–35 years) and its determinants has not yet been established in these areas and similar agro-ecological settings in Tanzania that this study seeks to address.

There are efforts to improve nutrition practices at household level and one way that has not been widely exploited is to encourage greater men’s participation in household food consumption decisions. To the best of our knowledge, the only empirical literature available is on how to involve men in chronic disease management in Mexico [17], maternal and newborn health[18] with limited studies on nutrition especially in SSA. One related study in Kenya found that men benefit more than women and children in terms of diet diversity because they often eat lunch and sometimes dinner away from home, thus increasing their chances of consuming other food items not usually available in their household [19]. Other project based observations show that men rarely participate in household nutrition decisions leaving this task to their wives, thereby leaving most women and children with smaller food portions and less nutritious meals compared to men[20]. Many interventions still focus on women neglecting the role of men in improving the household nutrition status. In this regard, this study contributes to this debate by empirically assessing men dietary patterns and their potential role in improving the nutritional status of all household members. The findings from this study initiates the policy dialogue on involving men in household consumption and nutrition decisions in order to develop innovative approaches to reduce undernutrition with an aim of increasing participation by men. The rest of the paper is organized as follows. In the next section, we describe the study area and sampling design, data collection and dietary diversity measurements. This is followed by the results and discussions while the last section is a summary of key findings and policy implications.

## Materials and methods

### Study area and sampling design

The study used cross-sectional survey data collected from Bahi District in Dodoma region and Mbarali District in Mbeya region of Tanzania. These districts are predominantly semi-arid in nature and experience low and erratic rainfall of about 500–650 mm per annum with most households relying on subsistence rain-fed agriculture. Introduction of irrigated agriculture is currently undertaken by the Tanzanian government, in collaboration with Competitive African Rice Initiative (CARI) program funded by Deutsche Gesellschaft für Internationale Zusammenarbeit (GIZ) and implemented in Mbarali and Bahi for growing paddy rice, maize and beans. In Tanzania, CARI began in 2015 with the aim of increasing rice productivity and complementary crops such as vegetables by enhancing farmers’ business skills and access to financial services while ensuring adoption of good agricultural practices. A baseline survey to understand the dietary diversity of the target households was conducted from December, 2015 to January, 2016. Field interviews were however avoided during the Christmas and the New Year festive periods of the survey to avoid biases, given high likelihood that household food consumption often does not reflect a typical diet. The study followed a multi-stage sampling procedure. In the first stage, Bahi and Mbarali districts were purposively selected due to their high levels of poverty and high malnutrition. In the second step, 20 villages were randomly selected from the list compiled by the District Agriculture Irrigation and Cooperatives Officers (DAICOs) and Competitive African Rice Initiative (CARI) program officials. Village is the lowest administrative unit in Tanzania. The lists of households in each village were obtained from DAICOs of the two survey districts and CARI. The third step involved a random sampling of the targeted households proportional to the number of the households participating in CARI program in the village, generating a total of 204 respondents. The response rate for the survey was 97 percent. From the total sample, 101 respondents were from Bahi and 103 respondents were from Mbarali District. As a complement, focus group discussions (FGDs) were conducted with men to qualitatively assess their dietary diversity and potential roles in combating micronutrient malnutrition challenges. The Critical Appraisal Skills Program (CASP) qualitative research checklist was used to ensure that the objectives of the research were appropriately addressed. The interview guide with questions were asked to the participants in a group and they were requested to answer together after collating thoughts. The village agricultural extension officers prepared a list of men participating in CARI program in the four villages (two per district) and from the list, a random sample of 10–15 men per village was drawn. In total, four FGDs were conducted prior to the main quantitative survey, two in each district and each lasted 60–90 minutes. A total of 50 men from Mbarali and Bahi District were covered during the focus group discussions. Respondents who participated in FGDs were not allowed to participate in the main household survey so as to prevent them from influencing the responses. The men who participated in the FGDs aged 29 to 58 years and were married with at least two dependents. The focus group discussions were conducted in two parallel sessions per district, covering different subject domains such as types of foods men consumed at home, types of foods men consumed away from home (breakfast, snack, lunch, snack, dinner and snack before bed time), men’s frequency of eating away from home, nutritional calendar of men and what types of foods are preferred by household members at different times and the frequency at which male respondents ate different food items per day etc (see more descriptions in S1 File).

### Data collection and measurement of dietary diversity

The dietary diversity score, developed by the Food and Agriculture Organization (FAO) of the United Nations, is a qualitative 24-hour recall of all the food and drinks consumed by the respondent (if measured at individual level) or any other household member (if measured at household level) [21]. The 24-hour recall period is subject to less recall error, less cumbersome for the respondents than seven or thirty day recall period, and also conforms to the recall time period used in many dietary diversity studies [22]. This was adopted given that Savy *et al*.,[23] suggested that a dietary diversity score calculated from a 24 hour recall is sufficient to describe households’, women’s and children’s nutritional status. Therefore, we computed household dietary diversity score (HDDS), children under five dietary diversity score (CDDS) [21, 24] and minimum dietary diversity for women (MDD-W)[25]. HDDS provides a snapshot of the economic ability of a household to access a variety of foods while CDDS and MDD-W reflects nutrient adequacy for the children and women respectively. A study conducted in Bangladesh, Mali, Mozambique, and the Philippines using standard dietary scores shows that an increase in dietary diversity in different age groups is related to increased nutrient adequacy of the diet for non-breastfed children [22], adolescents [26] and adults[7]. For the purposes of this study, women of child bearing age are defined as those between 15–35 years of age; also falling within the definition of a youth according to the Tanzanian government[27].

The previous women dietary diversity score did not propose a cut-off point for a dichotomous indicator, but we use MDD-W, which calculates the percentage of women achieving the minimum dietary diversity[25]. The minimum diverse diet for women has been defined as five food groups out of ten. World Health Organization (WHO) proposed a threshold of 4 foods out of seven for children between 6–23 months [28] but no consensus has so far been reached for situation related to the children under five. We therefore, adopt a cut-off of 4 or more food groups out of nine for children between 1–5 years old and have been used by Styn *et al*., [24]in South Africa. There are no cut-offs defined for the household diversity score [21].

To estimate dietary diversity, the questionnaire was administered to the person responsible for the household’s food preparation on the previous day. This person, in most cases, the mother responded to the questions about food consumed by children under 5 years old and a woman aged 15–35 years in the household recorded the food consumed to measure MDD-W. All the respondents gave their consent prior to participating in the study. The questionnaire used elicited information on the respondents’ dietary history in addition to being asked to recall all the different foods eaten and beverages taken in the previous 24 hours prior to the survey. A set of 12 food groups was used to estimate HDDS[21] and 10 food groups for MDD-W [25] and 9 food groups for CDDS[21, 24]. Previous research has shown that the food groups; ‘fats and oils, sugar/honey’ and ‘spices, condiments and beverages’ do not contribute to the micronutrient density of the diet and thus these food groups were not part of the MDD-W and CDDS[21, 25]. The food groups that were used in computing MDD-W indicator include (1) grains, white roots tubers, and plantains (2) Pulses (beans, peas and lentils) (3) Nuts and seeds (4) Dairy (5) Meat, poultry and fish (6) Eggs (7) Dark green leafy vegetables (8) Other vitamin A-rich fruits and vegetables (9) Other vegetables (10) Other fruits.

In order to accurately capture all the food groups consumed by children under five, additional probing was done for snacks eaten between main meals and special foods given to them by the mother or the person responsible for food preparation in the household. Responses relating to socio-economic characteristics of the households were also captured: gender of the household head, vegetable production, land size, access to credit and extension services, training received on food preparation and human nutrition among others. The information about men’s food consumption patterns were collected in the FGD sessions. The FGD were conducted by one moderator and three research assistants who recorded all the responses on paper. We prevented social desirability bias in the FGDs by clearly asking the questions and sometimes repeating more than once for the purposes of triangulation, asking the participants what they do (or would do) and not necessarily what they think should be done in relation to improving their dietary diversity.

### Data analysis

The DDS was calculated by summing the number of different food groups consumed by an individual or household over the 24-hour recall period. A score of 1 was given to each food group consumed. Maximum values of 12 points for HDDS, 10 points for MDD-W and 9 points for CDDS were obtained. Differences in household characteristics and dietary diversity scores between the two regions were assessed using independent t-test and chi-square (χ^2^) test. Factors influencing HDDS, MDD-W and CDDS was modelled using Poisson regression model. Cluster robust standard error adjustments were done in order to obtain robust results. Poisson regression is an appropriate tool to model count data (in this case dietary diversity score, which ranges from 0–12 for HDDS, 0–9 for CDDS and 0–10 for MDD-W)[29]. The explanatory variables included in the model are presented in Table 1. In addition, ordinary least squares (OLS) regression was also estimated to validate the study findings (S2 Table). OLS assumes that the outcome of interest (HDDS, MDD-W and CDDS) is normally distributed while poisson regression assumes that the outcome is Poisson distributed. Comparing results from the two models is important because originally linear regression model (OLS) was used when modelling count data [30]. The coefficient estimates of both models can be interpreted as semi-elasticities which represents percentage change in the dietary diversity score when the explanatory variable changes by one unit. STATA version 14 was used to analyse quantitative data. Content analysis method was used to manually analyse qualitative data and triangulated with key stakeholders’ reflection and learning workshop that was held in September, 2016. Data saturation point was reached upon conducting the fourth FGD and sampling more data could not lead to more information related to the research questions.

**Table 1:** Characteristics of sampled households in Bahi and Mbarali districts, Tanzania.

	(a)		(b)				
	Bahi District		Mbarali District		Total		Test^a^
Variable	Mean	SD^b^	Mean	SD^b^	Mean	SD^b^	a≠b
Household size (persons)	4.63	1.78	6.27	2.59	5.46	2.36	***
Years of education of household head	7.00	3.03	6.58	2.63	6.80	2.85	
Age of household head (years)	42.08	13.68	45.46	13.57	44.0	13.70	*
Female household head(percentage)	0.19	0.40	0.19	0.40	0.19	0.39	
Participated in training on food preparation and nutrition (proportion)	0.14	0.34	0.06	0.24	0.09	0.29	**
Agricultural land area (hectares)	1.99	2.27	1.39	1.66	1.69	2.00	**
Household grows vegetables (proportion)	1.00	0.00	0.19	0.39	0.59	0.49	***
Access to off-farm income (percentage)	0.78	0.42	0.15	0.35	0.46	0.50	***
Number of observations	101	103			204		

Categorical and continuous variables were tested using χ2 and t-tests adjusted for clustering, respectively.

^a^Asterisks denote the level of significance for a t-test/χ^2^-test of difference in means between the Districts, with *** p<0.01, ** p<0.05 and* p<0.10.

^b^SD = Standard deviation.

## Ethical considerations

The households involved in this survey were drawn from the CARI project participants and thoroughly informed about the objectives of the survey. The respondents were explicitly asked for their verbal informed consent to voluntarily participate in the study and recorded in the questionnaire. Prior to starting each interview, the study objectives were explained to the respondents. It was also clarified to them that the data collected would be kept strictly confidential, analysed anonymously and used for research purposes only. As a general requirement, we also obtained permission from District executive office to conduct the survey. This study was approved by the World Vegetable Centre’s Institutional Bio-safety and Research Ethics Committee (IBREC).

## Results

### Descriptive results

Descriptive statistics are presented in Table 1. Average household size was 4.6 persons in Bahi and 6.3 in Mbarali District. The average age of the household head was 42 years in Bahi and 45 years in Mbarali. Across both districts, 18% of households were female-headed, showing that the vast majority of households were led by males. On average, the household heads had six years of education. Moreover, only 11% of the households had been trained on food preparation and nutrition. Households in Bahi significantly owned more agricultural land (p<0.05) than those in Mbarali. About 20% of sampled households were growing vegetables in the past year (22% in Bahi, 18% in Mbarali). About 89% of the sampled households were active members of recognized farmers’ associations where knowledge about nutrition can be shared with other community members.

**Fig 1:**
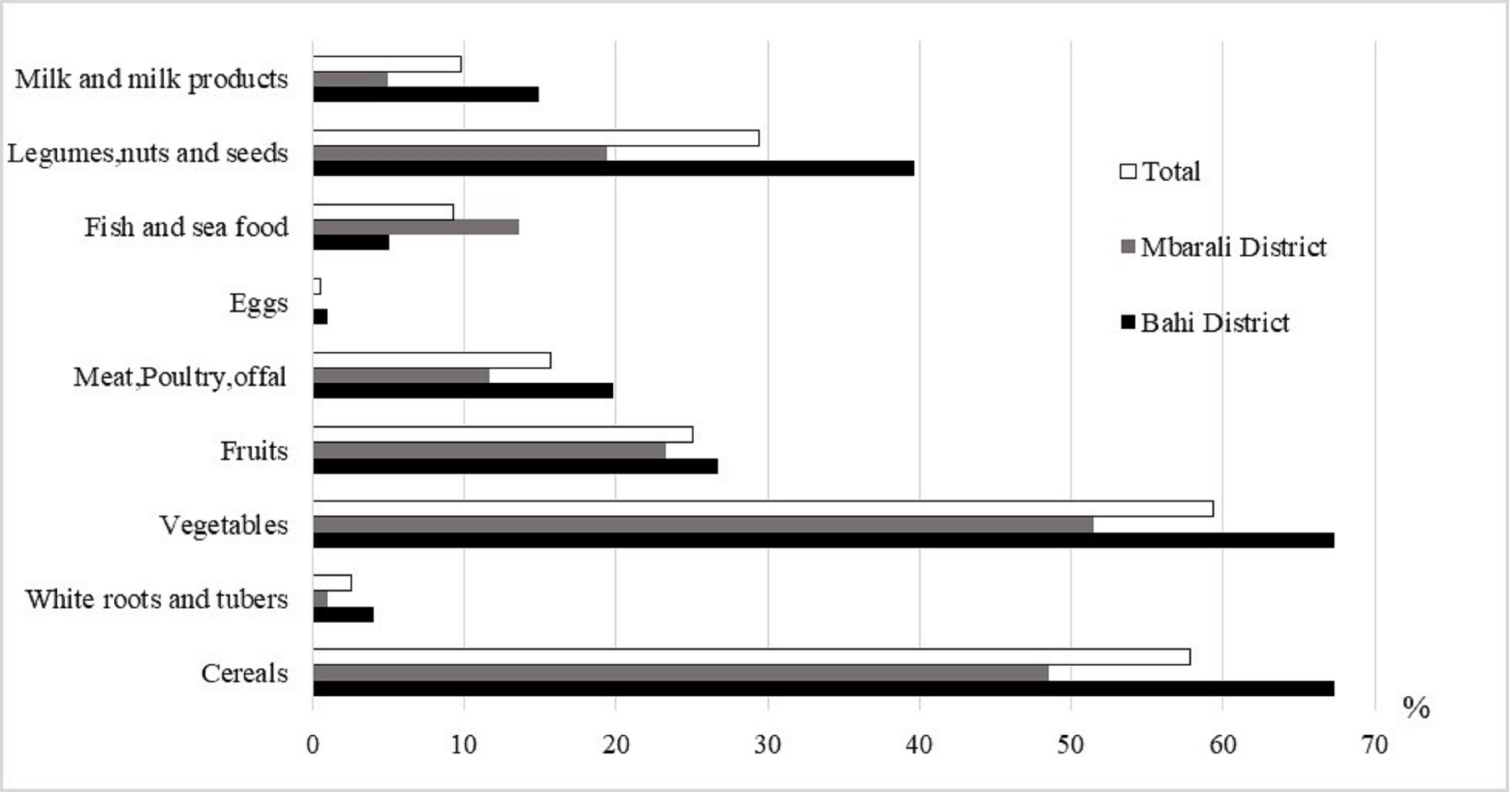
Percentage of children under 5 years who consumed each food group in 2015.

**Fig 2:**
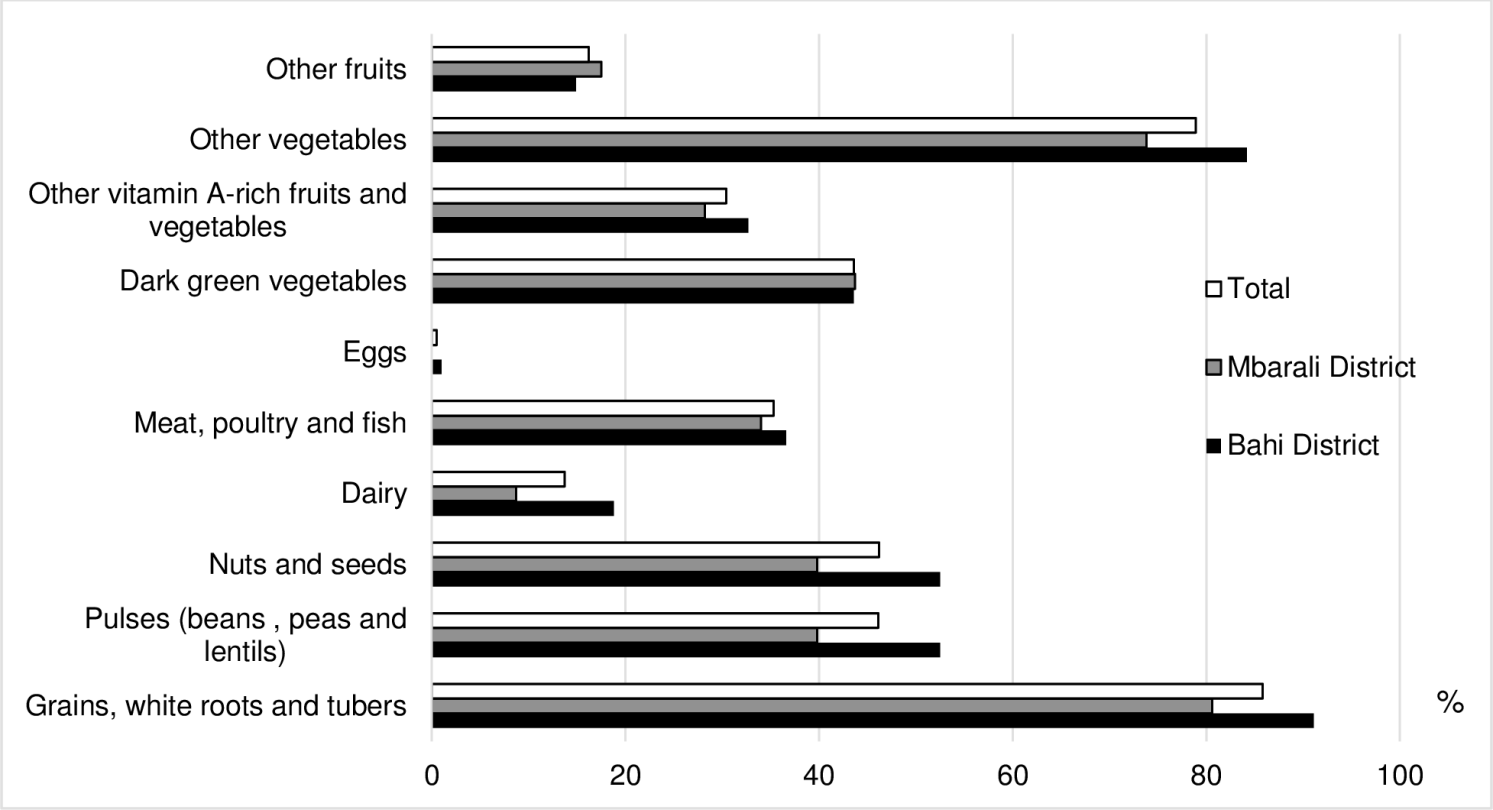
Percentage of women aged 15–35 years who consumed each food group in 2015.

The diet of the children and women mainly comprised cereals, vegetables, legumes, seeds and nuts (30–91 percent) (Figs 1 and 2). Meat-based products (beef, poultry and fish), milk and eggs were rarely consumed by children and women. Cereals and white roots and tubers were consumed by 86% of the women.

**Fig 3:**
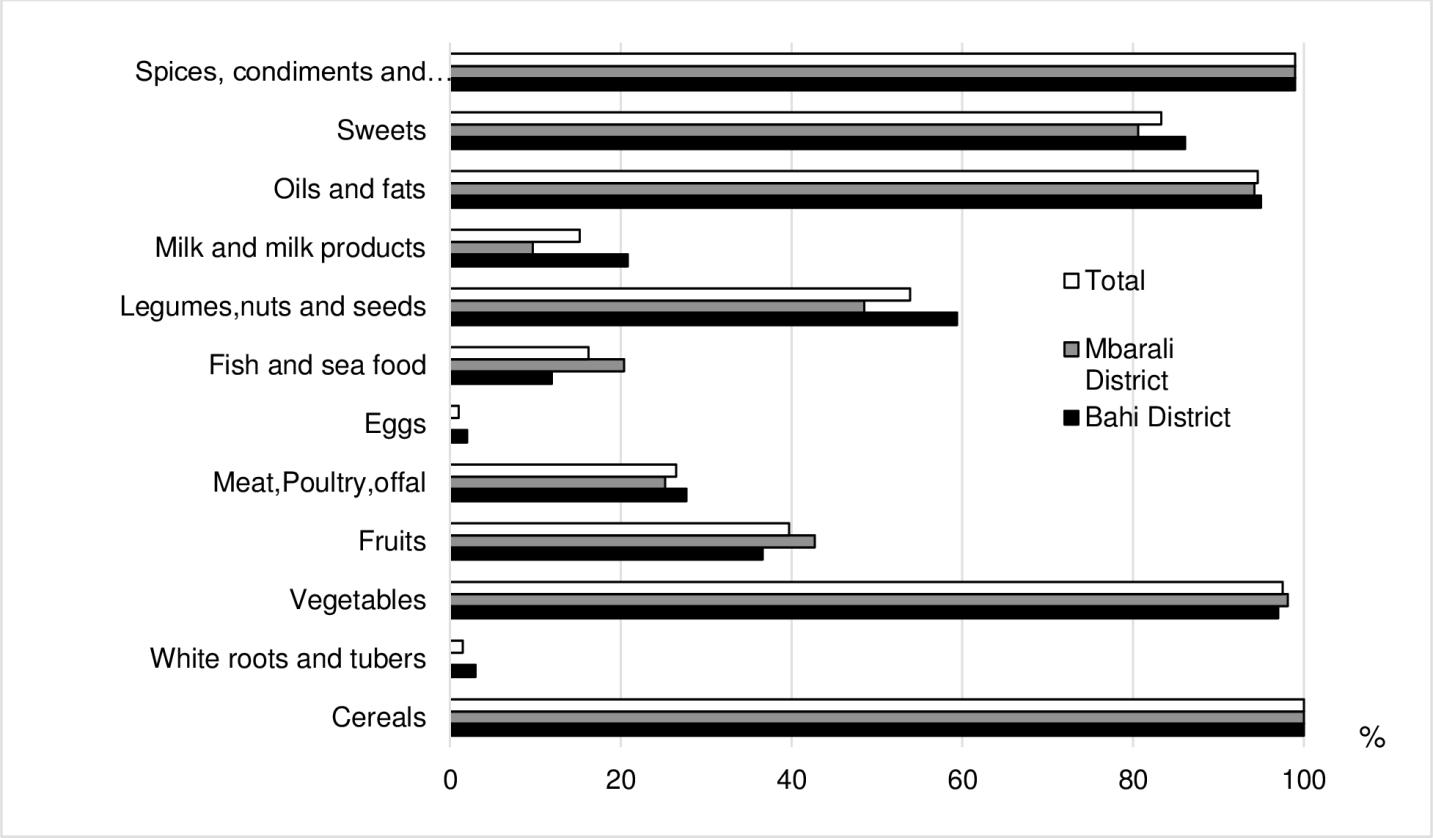
Percentage of households who consumed each food group in 2015.

The food groups commonly consumed by the households were cereals (100%); spices, condiments and beverages (99%); vegetables (98%); oils and fats (95%); sweets (83%); legumes, nuts and seeds (54%); and fruits (39%) (Fig 3). Meat-based products (i.e. poultry, offal, fish, etc.), eggs, milk, and milk products were rarely consumed by many households. For example, less than one percent of the households consumed eggs, while less than 16% consumed fish/seafood milk and milk products (Fig 3).

### Dietary diversity score of women, children under five and the household

The average HDDS was 6.28, with 6.39 and 6.18 in Bahi and Mbarali respectively. On average, households consumed six food groups over the preceding 24-hour recall period. (Table 2). The results further show that children and women consumed two and four food groups, respectively. Disaggregating by district, children under five in Bahi (2.46) displayed higher dietary diversity score (p<0.1) than those in Mbarali (1.74).

**Table 2:** Average household dietary diversity.

Dietary Diversity Scores	Bahi (a)	Mbarali (b)	MHH^b^ (c)	FHH^b^ (d)	Total	Test (t-value)	Cohen *d*	Test (t-value)	Cohen *d*
	Mean	Mean	Mean	Mean	Mean	a≠b		c≠d	
HDDS^a^	6.39	6.18	6.26	6.38	6.28	1.18	0.2	-0.52	0.1
CDDS^a^	2.46	1.74	2.22	1.58	2.09	1.62*	0.4	1.97**	0.4
MDD-W^a^	4.28	3.66	4.15	3.20	3.97	2.23**	0.3	2.75***	0.5
Sample size (N)	101	103	164	40	204				

^a^CDDS = Children’s Dietary Diversity Score (1–5 years old)

MDD-W = Minimum women dietary diversity for women (15–35 years old); HDDS = Household dietary diversity score.

Asterisks denote the level of significance for a t-test of difference in means adjusted for clustering

*** p<0.01.

**p<0.05.

*p<0.1.

^b^FHH-Female headed household; MHH-Male headed household.

Cohen *d* estimates the effect size.

**Fig 4:**
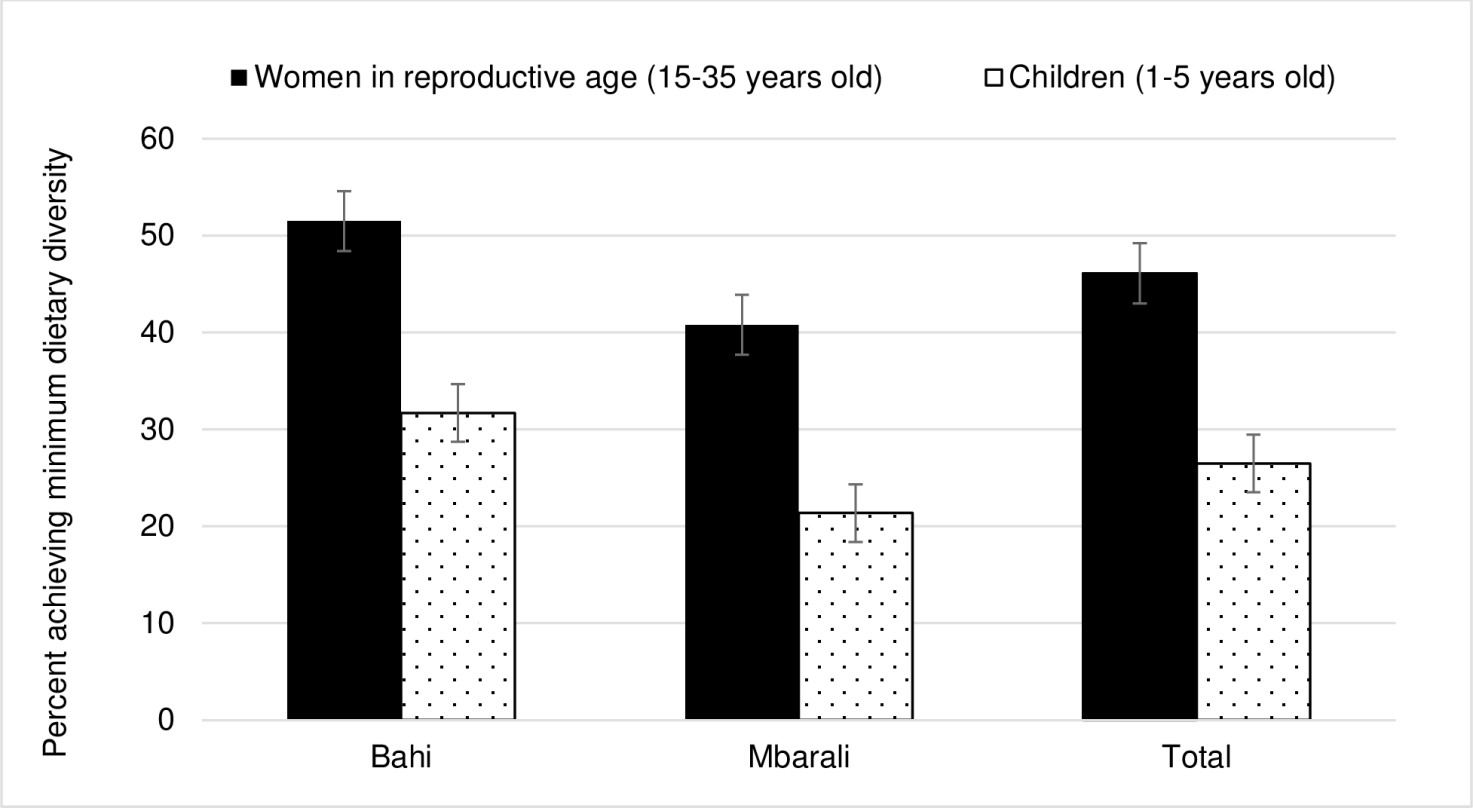
Percentage of women (15–35 years) and children (1–5 years) achieving minimum dietary diversity in 2015.

Dietary diversity scores for children (1.6) and minimum dietary diversity score for women (3.2) were lower in female-headed households compared to male-headed households. However, HDDS is similar in both types of households with them consuming foods from six food groups. The percentage of children and women achieving minimum dietary diversity is presented in Fig 4. Averagely, 46% of women achieved minimum dietary diversity, thus are more likely to have higher micronutrient intake than the 54% of women who did not. Similarly 26% of the children achieved minimum dietary diversity; four food groups out of nine.

In order to further assess dietary diversity, their categories were formulated namely; low dietary diversity category (≤3 food groups); medium diversity category (4 to 5 food groups) and high diversity category (≥6 food groups) for household, children and women. On average, 31% of the women were in the lowest dietary diversity category (consuming less than or equal to three food groups) while 73% of children consumed three food groups or less (S1 Table). When considering the whole household, about 80% (78% in Bahi and 82% in Mbarali) had high dietary diversity (at least one person consumed six or more food groups) (S1 Table).

### Determinants of dietary diversity

Gender and education level of the household head and size of agricultural land influenced HDDS (p<0.01) (Table 3). Education of the household head was positive and significantly influenced the household’s, children’s and women’s dietary diversity. CDDS and MDD-W decreased with the increase in the age of the household head. Households headed by women had significantly higher household dietary diversity (p<0.05).

**Table 3:** Determinants of dietary diversity scores.

Variables	(1)		(2)		(3)	
	HDDS^a^		CDDS^a^		MDDW^a^	
	Coefficient	P>|z|	Coefficient	P>|z|	Coefficient	P>|z|
Household size (persons)	-0.002	0.775	0.047	0.172	0.033	0.121
	(0.006)		(0.034)		(0.021)	
Years of education of household head	0.016***	0.002	0.061**	0.012	0.022*	0.080
	(0.005)		(0.024)		(0.013)	
Age of household head (years)	-0.001	0.457	-0.016***	0.001	-0.007**	0.018
	(0.001)		(0.005)		(0.003)	
Gender of the household head (= 1 if female)	0.051**	0.019	-0.243	0.148	-0.162	0.245
	(0.023)		(0.168)		(0.140)	
Participated food and nutrition training (= 1 if yes)	0.099**	0.016	0.099	0.617	0.082	0.457
	(0.041)		(0.198)		(0.110)	
Agricultural land area (hectares)	0.016***	0.000	-0.013	0.377	0.030***	0.000
	(0.004)		(0.016)		(0.008)	
Whether household grows vegetables (= 1 if yes)	0.044	0.280	-0.074	0.858	0.339**	0.020
	(0.041)		(0.417)		(0.146)	
Access to off-farm income (= 1 if yes)	0.004	0.941	0.129	0.171	0.054	0.573
	(0.052)		(0.095)		(0.096)	
District (= 1 if Mbarali)	0.036	0.484	-0.345	0.369	0.137	0.270
	(0.052)		(0.384)		(0.125)	
Constant	1.670***	0.000	0.927*	0.089	1.003***	0.000
	(0.072)		(0.545)		(0.234)	
Variance inflation factor (VIF)^b^	1.84		1.84		1.84	
Observations	204		204		204	

^a^Robust standard errors in parentheses. Cluster adjusted by villages.

Asterisks denote the level of significance at

*** p<0.01.

** p<0.05.

* p<0.1.

CDDS = Children’s Dietary Diversity Score (1–5 years old); MDD-W = Minimum Dietary Diversity Score for women (15–35 years old); HDDS = Household Dietary Diversity Score.

^b^VIF obtained after regress since collinearity is a property of the independent variables only. Similar results are reported in S2 Table.

Land is an important factor for diet diversity and households owning larger areas of agricultural land had higher HDDS and MDD-W (Table 3). Most of the households in Bahi and Mbarali districts derived more than 90% of their incomes from agriculture, thus land is important for food production and dietary diversity. Households growing vegetables had significantly higher MDD-W, yet in Mbarali only 19% of the households grew vegetables (Table 1). To test the robustness of the results, we re-estimated the determinants of dietary diversity using OLS regressions (also explained in the method) and the significant factors did not change (S2 Table) giving confidence in the Poisson results.

### Men’s dietary patterns and their potential role in household nutrition security

This section presents FGD results. The food consumption among men varied depending on the season, and differed between the seasons when food is in surplus (April-August) and in shortage (September-March). During the season when food is in surplus, households could easily access all food categories by selling their harvested produce (i.e., rice, maize, and beans) to purchase other food items such as meat, milk, sugar, and fish, among others. Households with income obtained from selling produce were able consume black or milk tea with either rice, ugali boiled tubers or nuts as breakfast and occasionally served either rice, ugali with fish or meat for lunch and dinner. Ugali is stiff porridge made from either maize flour, sorghum or millet flour. There were long periods of food shortage in Bahi and Mbarali beginning from mid-November to March, to the extent that some households had only one meal a day, mainly carbohydrate-based foods such as ugali, cassava and rice.

From the FGD it emerged that men also dominantly took black or milk tea with rice or *mandazi* and *chapati* for breakfast and rice or ugali with fish or meat for lunch and dinner from food vendors during food shortages, while rice or ugali with vegetables were served at home (Table 4). *Chapati* and *mandazi* are wheat based doughnuts usually not made at home because they take a long time to prepare. These products are an example of frequently consumed affordable foods prepared outside of the home with a high fat content. This situation has resulted in a gap between food consumed at home and food eaten away from home. Most men (more than half of participants) consumed food outside the home. Foods commonly consumed from outside included meat or fish, chips made from tubers plus eggs or roasted meat, milk tea and *chapati*. Majority of men have access to food twice: at home, and outside in a restaurant or hotel before going home, as indicated by all participants. One participant from Bahi said, *“If I can only afford a half kilogram of meat in the bar; will it be enough for a household of 6 members*? *You will agree with me that it will be my option to consume it myself so that I get enough energy to feed my family*.*”* In addition, men do not take food home because of cultural beliefs. For example, one participant from Mbarali said, “*It is a custom in our area to be perceived as if you are controlled by your wife if you take home food items such as vegetables or meat*.*”*

**Table 4:** Male dietary patterns during food shortages in Mbarali and Bahi districts.

	Mbarali		Bahi	
	Eat at home	Eat from outside	Eat at home	Eat from outside
Breakfast	^a^Milk or black tea or porridge +kiporo^**b**^	Milk tea+ *chapati*	Porridge or black tea +kiporo^**b**^	Milk tea+*chapati*
Lunch	Rice+vegetables +legumes	Rice +meat/fish+fruit	Ugali+vegetables+legumes	Rice +meat/fish+ vegetables
Dinner	Ugali+vegetables+legumes	Ugali+meat/fish+fruit	Rice+vegetables+legumes	Ugali+meat or fish+vegetables
Snacks	(a) Fruits *(mangoes)* (b) Roasted groundnuts	(a) Chips +roasted meat/eggs (b) Local beer *(made from bamboo juice)* (c) Soft drinks like *soda/juice* &*bread/biscuits*	(a) Boiled groundnuts or bambara nuts (b) Fruits *(dates*, *mangoes & baobab fruits)*	(a) Chips + roasted meat/fish/fried eggs (b) Local beer *(made from maize/sorghum flour*) (c) *Soft drinks like soda/juice &bread/biscuits*

^a^In Mbarali, livestock keepers inhabit the area in search of pastures and farmers often buy milk from them.

^b^*Kiporo* is the left-over food from dinner such as ugali, rice etc.

Most households ate ugali or rice with leafy vegetables or beans throughout the year without access to protein rich foods such as fish which men accessed outside the household. Most participants opined that the risk of inadequate nutrition increases with increased frequency of food consumption away from home by the male household members particularly in times of food shortages. During food surplus seasons, men often sold produce to get cash for purchasing food prepared outside the home mostly in restaurants and hotels instead of purchasing food for the whole household. The participants pointed out that cash received by women was mainly spent on food and wellbeing of the children.

Consumption of important food items such as vegetables, fruits, meat, fish and eggs was low, yet lack of consumption contributes to malnutrition (S3 Table). For example, meat products were either not consumed at all or consumed once per day, particularly during the food shortage period compared to starchy foods (i.e., consumed 3–5 times per day). It emerged that alcohol was mainly consumed during food shortage periods as a way of dealing with stress.

## Discussion

This paper estimated factors influencing dietary diversity of the household, children under five years, and women; and qualitatively assessed male dietary patterns and their potential role in improving their household nutritional status in Tanzania. Findings show that the diet of households lacked diversity, and the intake of foods from animal sources (i.e., meat, poultry and offal, fish) was low, especially among children under five years and women of child bearing age (15–35 years). Most children consumed foods from 3 or fewer food groups on the day prior to the survey. On average, 46% and 26% of women and children respectively, achieved minimum dietary diversity. Vegetable production emerged as an important activity for improving dietary diversity, particularly for women. In addition, gender and education of the household head, food and nutrition training, education and farm size were important determinants of dietary diversity. From FGD results, men can contribute to improved food and nutrition security by reducing their consumption of food away from home particularly, during food shortages and using savings to purchase nutritious food for the entire household. These summarized results are discussed in detail below.

### Dietary diversity and its determinants

Our study confirms that the diet of the children and women mainly comprised cereals, roots and tubers, vegetables, legumes, nuts, seeds and fruits. These results are similar to findings reported in rural areas of Ethiopia, Burkina Faso and Tanzania [8, 31, 32]. These findings equally supports the notion that women and children under five years are more likely to be disadvantaged than men and are at risk of poor health [33] due to high consumption of carbohydrate-rich staple crops with few animal products, fruits, and vegetables. Women of child bearing age often require energy, protein and micronutrients such as iron, particularly when they are expectant and breast feeding [1, 34, 35]. Few households consumed eggs, milk and milk products due to inability to purchase animal products which are often sold in the local market and rarely consumed at home. Several studies indicate that increasing dietary diversity is crucial for increasing women’s and adolescents’ ability to perform well at school and to be ready to take up economic opportunities [1, 36] thus it requires much more focus by all stakeholders implementing food and nutrition security programs.

Dietary diversity scores for children and minimum dietary diversity scores for women are lower in female-headed households compared to male-headed ones. The relatively low diversity scores for children (CDDS) and for women (MDD-W) can be attributed to the high poverty levels among women-headed households, since in most rural households, women are responsible for family nutrition. However, the current findings does not show that poor households have low dietary diversity. A previous study using 14 food groups reported that women who are household heads had higher women dietary diversity scores than those from the male heads in Kongwa, Muheza and Singida Districts in Tanzania [32]. However, our study adopted new method of measuring women dietary diversity (MDD-W) by using 10 food groups as opposed to usual 9 food groups. Most children and women consumed three or fewer food groups characterized with limited consumption of foods from animal sources confirming findings from Burkina Faso[8]. Traditionally in several parts of Tanzania children are mainly weaned on starch based foods and few of them are given meat and vegetables at this stage. For example, the most common weaning foods observed in rural areas of Tanzania are either maize porridge mixed with milk or cow’s milk which has led to persistent high rate of child malnutrition [37].

In terms of socio-economic characteristics presented in Table 1, Bahi compared to Mbarali District had more household members, majority participated in nutrition and food preparation training, larger land sizes, accessed more off-farm income sources and grew vegetables. The plausible reason for this is because Bahi is closer to Dodoma city–Tanzania’s capital city which provides the population with the market for their products and are able to access more off-farm jobs to supplement farm income. Low dietary diets observed in Mbarali District, could be due to the general low agricultural diversity in the district where rice growing is the main economic activity dominated by large scale farmers. Small-scale farmers targeted in the present study are less involved in paddy rice production in Mbarali District.

We estimated the factors influencing dietary diversity by comparing Poisson and Ordinary least squares (OLS) regressions estimates to ensure robustness of the results. Findings show that education of the household head contributed to household dietary diversity. Education is likely to have an impact on the household’s nutritional knowledge and skills to conceptualize and use nutritional promotional messages, which consequently contribute to better dietary diversity[38]. A previous study in Morogoro in Tanzania reported that households who were provided with nutritional education improved the quality of their household diets[39]. Households headed by women significantly had higher household dietary diversity scores. This could be because in SSA, women- controlled income often has greater benefits for the nutrition, health and well-being of all household member, especially children, than men-controlled income [40–43]. Rural women’s income tend to come frequently from petty trades though in smaller amounts thus is readily spent on household daily subsistence needs than men’s lumpier seasonal income which is likely to be spent on more expensive items [41]. Land is important for both household and women dietary diversity implying that it would be more beneficial to promote equitable land distribution and provide women with land for food production, as a measure to increase food and nutrition security among rural communities. Size of the land owned does not predict the children dietary diversity probably because, adults consume lots of food products directly from the farm compared to children below five who are sometimes provided with special purchased foods such as infant cereals (e.g., *Weetabix and Cerelac*). Vegetable production influences dietary diversity particularly that of women. Vegetables are major sources of micronutrients, vitamins and minerals, yet few households (less than 20% in Mbarali district) grew them (see Table 1). Kending *et al*., [32], also reported in rural Tanzania that women who cultivated or collected vegetables in all seasons had higher dietary diversity compared to those who did not. This clearly shows that vegetables form part of balanced diet and could also be purchased from the local market in addition to what is produced by the household.

### Men’s dietary patterns and their potential role in household nutrition security

Foods commonly consumed from outside by men in the area of study include meat or fish, potato chips, eggs and roasted meat, milk tea and *chapati* during food shortage while at home rice or ugali with vegetables are usually served for dinner (Table 4). These findings confirm those from Kenya that food consumed outside the home contributes more to the diets of men [19]. Therefore, male dietary patterns depend on what is consumed outside the home, which contributes to the gap in dietary diversity of the members of the same household. This is likely to affect some household members negatively, particularly children under five and women, who are more susceptible to undernutrition. Our study contributes to the claims that not only are women in developing countries more likely to be malnourished than men, but also have less access to nutritious food than men. It is worth noting that sociocultural traditions and differences in household work patterns can potentially increase women's chances of being more malnourished. Thus, men should work together with women to plan how the farm can produce healthy food for the family and provide nutritious food that benefits the entire household. From the FGDs, it was evident that ensuring household food security requires men to be adequately sensitized to avoid selling all their farm produce during surplus periods. This requires thoughtful planning and consultation with local leaders, men and women to identify the most appropriate method to implement community-based sensitization programs. A study by Ochieng *et al*., [44] found that such community-based sensitization programs were useful in increasing consumption of traditional African vegetables and impacted positively on dietary diversity of children and women in the Arusha region of Tanzania. Furthermore, agricultural development programs should encourage both husband and wife in most nutritionally vulnerable households to collectively participate in resource allocation for its production and consumption decisions[45], thereby helping to improve the nutritional status of the household.

Discussions with men concluded that women were more likely than men to spend their income on food and child welfare leading to better household nutritional outcomes. However, women’s efforts in SSA are often thwarted by constraints such as access to production resources, limited access to credit as well as doubling as mothers and farm managers [41]. Men’s propensity to excessively consume food (e.g. meat products that may have not been prepared at home) away from home, contributes to the decline of food and nutrition security of other household members. This suggests that the challenges of malnutrition can be reduced if men would join hands with their women counterparts to provide food for the family especially during food shortages.

## Study limitations

The present study was conducted in two districts in Tanzania thus the results cannot be generalized at the national level because the sample size is not representative of the whole country. Further research work with larger sample sizes is recommended. Additionally, the cross-sectional nature of the data does not allow examining causality in the relationship between dietary diversity and socio-economic factors. The study qualitatively measured dietary patterns of men thus, there is need to quantitatively measure the men’s dietary diversity and compare it with those of other household members. Despite the limitations outlined, the methods adopted in this study had a number of advantages as follows. First, the sampling process was done together with the local and CARI program administrators and survey administered by qualified researchers from World Vegetable Center, Eastern and Southern Africa in Tanzania. Secondly, two modelling approaches: Poisson and multiple regression (OLS) have been used making the results robust and reliable. Thirdly, it provides a useful base for further research on how to involve men in nutrition matters. Finally, the findings contribute to the limited body of knowledge on dietary diversity and the potential role of men in improving household nutrition in Tanzania and beyond.

## Conclusions

Food and nutrition insecurity remains a complex problem, mainly in rural areas in less developed countries such as Tanzania. Our study revealed that the diet among households lacked diversity, and that the intake of foods from animal sources (meat, poultry and offal, fish and seafood) was low, especially among the children and women. Women and children access a less diverse diet, since 46% and 26% achieved minimum dietary diversity respectively. Children under five and women in female-headed households had significantly low dietary diversity scores compared to those in the male-headed households. Vegetable production emerged as an important subsector in improving household dietary diversity, particularly for women. Additionally, gender and education of the household head, training on food and nutrition and size of land owned contribute to improved dietary diversity.

Men can contribute towards improving household nutrition security by reducing their consumption of food away from home, particularly during food shortages, and using the money saved to purchase nutritious food for the household. Male farmers also could set aside a part of the produce harvested for household consumption rather than market sale. These actions could potentially reduce any form of malnutrition among children and women. Because most households depend on agriculture, awareness creation should focus on improving the production of vegetables and consumption of foods with superior micronutrient density for improved nutrition. Thus, there is a need to support community-based sensitization programs to provide information on food preparation, human nutrition, and the importance of growing vegetables for household consumption. Such programs should focus on encouraging men to actively participate in providing diversified diet to their households.

## Supporting information

S1 Table Percentage of households, children and women in different dietary diversity categories. (DOCX)

S2 Table OLS regression results for determinants of dietary diversity. (DOCX)

S3 Table Men’s frequency of consuming different food categories. (DOCX)

S1 File Focus group discussions (FGD) interview guide. (DOCX)
