# Supplementary material for: Determinants of dietary diversity and the potential role of men in improving household nutrition in Tanzania
Source: PLoS One. 2017 Dec 12;12(12):e0189022. doi: 10.1371/journal.pone.0189022 (PMC5726653; doi:10.1371/journal.pone.0189022)
Supplement: S1 Table — (DOCX) [file pone.0189022.s001.docx]

### S1 Table. Percentage of households, children and women in different dietary diversity categories

|  | Dietary diversity category | Bahi | Mbarali | Overall |
| --- | --- | --- | --- | --- |
| Household |  |  |  |  |
|  | Lowest DDS (≤ 3 food groups) | 0.0 | 3.0 | 1.5 |
|  | Medium DDS (4 and 5 food groups) | 22.3 | 14.9 | 18.6 |
|  | High DDS (≥ 6 food groups) | 77.7 | 82.2 | 79.9 |
| Children (1-5 years old) |  |  |  |  |
|  | Lowest DDS (≤ 3 food groups) | 68.3 | 78.6 | 73.5 |
|  | Medium DDS (4 and 5 food groups) | 27.7 | 20.4 | 24.0 |
|  | High DDS (≥ 6 food groups) | 4.0 | 1.0 | 2.5 |
| Women (15-35 years) |  |  |  |  |
|  | Lowest DDS (≤ 3 food groups) | 24.8 | 60.2 | 31.4 |
|  | Medium DDS (4 and 5 food groups) | 53.5 | 38.8 | 48.5 |
|  | High DDS (≥ 6 food groups) | 21.7 | 1.0 | 20.1 |
| Sample size (N) | | 101 | 103 | 204 |

DDS=Dietary Diversity Score.
