# Supplementary material for: Determinants of dietary diversity and the potential role of men in improving household nutrition in Tanzania
Source: PLoS One. 2017 Dec 12;12(12):e0189022. doi: 10.1371/journal.pone.0189022 (PMC5726653; doi:10.1371/journal.pone.0189022)
Supplement: S2 Table — (DOCX) [file pone.0189022.s002.docx]

S2 Table. OLS regression results for determinants of dietary diversity

| VARIABLES | (4) | | (5) | | (6) | |
| --- | --- | --- | --- | --- | --- | --- |
|  | HDDS^a^ | | CDDS^a^ | | MDDW^a^ | |
|  | Coefficient | P>\|z\| | Coefficient | P>\|z\| | Coefficient | P>\|z\| |
| Household size (persons) | -0.010 | 0.802 | 0.091 | 0.209 | 0.128 | 0.171 |
|  | (0.033) |  | (0.070) |  | (0.090) |  |
| Years of education of household head | 0.101^***^ | 0.007 | 0.110^**^ | 0.042 | 0.078* | 0.096 |
|  | (0.033) |  | (0.051) |  | (0.045) |  |
| Age of household head (years) | -0.004 | 0.468 | -0.030^***^ | 0.000 | -0.026^**^ | 0.021 |
|  | (0.006) |  | (0.007) |  | (0.010) |  |
| Gender of the household head ( 1 if female) | 0.337^**^ | 0.026 | -0.382 | 0.175 | -0.514 | 0.255 |
|  | (0.139) |  | (0.271) |  | (0.438) |  |
| Participated food and nutrition training (1 if yes) | 0.659^**^ | 0.041 | 0.217 | 0.648 | 0.347 | 0.495 |
|  | (0.300) |  | (0.466) |  | (0.499) |  |
| Agricultural land area (hectares) | 0.113^***^ | 0.001 | -0.032 | 0.290 | 0.147^***^ | 0.000 |
|  | (0.030) |  | (0.029) |  | (0.034) |  |
| Whether household grows vegetables (1 if yes) | 0.279 | 0.315 | -0.173 | 0.812 | 1.393^**^ | 0.039 |
|  | (0.270) |  | (0.716) |  | (0.628) |  |
| Access to off-farm income (1 if yes) | 0.0260 | 0.939 | 0.280 | 0.185 | 0.216 | 0.601 |
|  | (0.334) |  | (0.204) |  | (0.406) |  |
| District (1 if Mbarali) | 0.233 | 0.505 | -0.687 | 0.315 | 0.665 | 0.273 |
|  | (0.343) |  | (0.667) |  | (0.589) |  |
| Constant | 5.220^***^ | 0.000 | 2.572*^*^ | 0.020 | 2.417^**^ | 0.024 |
|  | (0.459) |  | (1.017) |  | (0.983) |  |
| Observations | 204 |  | 204 |  | 204 |  |

^a^Robust standard errors in parentheses and cluster adjusted by villages. Asterisks denote the level of significance at *** p<0.01, ** p<0.05, * p<0.1. CDDS=Children’s Dietary Diversity Score (1-5 years old); MDD-W=Minimum Dietary Diversity Score for women (15-35 years old); HDDS=Household Dietary Diversity Score.
