# Supplementary material for: Determinants of dietary diversity and the potential role of men in improving household nutrition in Tanzania
Source: PLoS One. 2017 Dec 12;12(12):e0189022. doi: 10.1371/journal.pone.0189022 (PMC5726653; doi:10.1371/journal.pone.0189022)
Supplement: S3 Table — (DOCX) [file pone.0189022.s003.docx]

S3 Table. Men’s frequency of consuming different food categories

| Food Group | | **Bahi District** | **Mbarali District** |
| --- | --- | --- | --- |
|  |  | *Number of times per day* | *Number of times per day* |
| 1 | Starch^a^ | 3 to 5 times | 3 to 5 times |
| 2 | Fruits | 1 to 2 times | 1 to 2 times |
| 3 | Vegetables | 1 to 2 times | 1 to 2 times |
| 4 | Dairy (Milk, yogurt) | 1 to 2 times | Once or never |
| 5 | Meat, fish, poultry and eggs | Once or never | 1 to 2 times |
| 6 | Fat (butter, margarine, oil) | 3 to 5 times | 3 to 5 times |
| 7 | Sweets (candy, cake, soda etc) | 3 to 5 times | Once or never |
| 8 | Alcoholic drinks^b^ | 6 to 8 times | 1 to 2 times |

^a^Ugali, rice, wheat products like bread, roll, pasta, noodles and potato.

^b^Alcohol: Local beer and industrially brewed beer that are readily available in the villages, although local beer is affordable and consumed more than industrially brewed beer
