# Supplementary material for: Determinants of dietary diversity and the potential role of men in improving household nutrition in Tanzania
Source: PLoS One. 2017 Dec 12;12(12):e0189022. doi: 10.1371/journal.pone.0189022 (PMC5726653; doi:10.1371/journal.pone.0189022)
Supplement: S1 File — (DOCX) [file pone.0189022.s004.docx]

## S1 File. Focus Group Discussions guidelines

Male dietary pattern focus group discussion (FGD) guide in Bahi and Mbarali Districts

**INSTRUCTIONS:**

*Number of FGDS: 2 per district*

*Number of participants: Key male informants of 10-15 men randomly sampled.*

*Purpose: To assess the male dietary patterns*

*Note that information regarding the nutritional calendar should be gathered during the focus group discussions*

**District________________ Location of the FGD (Village) ____________ Number of participants____________Date __________**

**SECTION A: This section seeks to assess *the male dietary patterns in the district. Ask each question all FGD Participants, allow them to interact and record the responses***

1. Which are the most common foods categories in this district? (*Facilitators should* *write down each food composition being mentioned by participants)*

2. (a) Which foods categories do men regularly eat in your location (village/district)?

( b) Which meals do men regularly eat outside their households ? At breakfast, Lunch, Dinner and Snacks?

3. (a) When do men take snacks most of the time and why? *(Record all different opinions and record all the comments made).*(for example in the morning, afternoon, evening, late night, throughout the day)

(b) What are men’s five (5) top favorite snack foods (*write all the foods/snacks mentioned)*?

4. Do men usually eat out or order food from outside the household *(allow for discussion and take notes)?*

______ Yes(why) ______ No (why); How often? (Daily, Weekly, Monthly etc).

5. (a) How are men’s food usually prepared? (*Allow for discussions).*

(b) Are men’s favorite foods in your location usually prepared and consumed at home or outside home?

6. In your opinion, how should men be engaged in providing food for their family? Are there any challenges? (*Allow for discussions and record everything*).

7. How many times each day do men have the following food items *(Allow for discussions as you record different views)*

| s/n. | Food groups |  | Never | Less than 1 | 1-2 times | 3-5 times | 6-8 times | 9-11 times |
| --- | --- | --- | --- | --- | --- | --- | --- | --- |
| 1 | Cereals | Rice, maize, sorghum, millet, bread, noodles, porridge |  |  |  |  |  |  |
| 2 | White Roots, Tubers And Plantain | Non-grain starchy staples: cassava, white potatoes, white yams, non-orange sweet potatoes, turnip, lotus root, taro root, arrow root, cooked or roasted green bananas |  |  |  |  |  |  |
| 3 | Vitamin A Rich Vegetables And Tubers | Pumpkin, carrot, squash, orange tomato, orange- or dark-yellow sweet potato, red sweet pepper |  |  |  |  |  |  |
| 4 | Dark Green Leafy Vegetables | Spinach, basil, morning glory, coriander leaf, amaranth, pumpkin shoots, mustard leaf, kale, chayote shoot, chard, broccoli, traditional or wild leafy vegetables |  |  |  |  |  |  |
| 5 | Other Vegetables | Onion, leek, tomato, fresh bean, eggplant, chayote fruit, lufa, gourds, cabbage, cucumber, okra, raw papaya, cauliflower, celery, fresh corn |  |  |  |  |  |  |
| 6 | Vitamin A Rich Fruits | Ripe mango, cantaloupe, ripe papaya, passion fruit, persimmon |  |  |  |  |  |  |
| 7 | Other Fruits | Other fruits (pineapple, guava, , orange, banana, avocado, etc.), including wild fruits and 100% fruit juice made from these |  |  |  |  |  |  |
| 8 | Organ Meat | Liver, kidney, heart, stomach, intestines (matumbo) or other organ meats or blood-based foods |  |  |  |  |  |  |
| 9 | Flesh Meats | Beef, pork, goat, sheep, game, chicken, duck, other birds, insects |  |  |  |  |  |  |
| 10 | Eggs | Eggs from chicken, duck or any other egg |  |  |  |  |  |  |
| 11 | Fish And Seafood | Fresh, smoked or dried fish or shellfish |  |  |  |  |  |  |
| 12 | Legumes, Nuts And Seeds | Dried beans, dried peas, mungbean, nuts, seeds or foods made from these (eg., peanut butter, legume sprouts) |  |  |  |  |  |  |
| 13 | Milk And Milk Products | Milk, cheese, yogurt or other milk products |  |  |  |  |  |  |
| 14 | Oils And Fats | Oil, fats or butter added to food or used for cooking |  |  |  |  |  |  |
| 15 | Sweets | Sugar, honey, sweetened soda or sweetened juice drinks, sugary foods such as chocolates, candies, cookies and cakes |  |  |  |  |  |  |
| 16 | Spices, Condiments, Beverages | Spices (chili, salt), condiments (fish sauce, sauce, hot sauce), coffee, tea, alcoholic beverages, local brew |  |  |  |  |  |  |

**SECTION B: This Section aims to analyze men’s knowledge of their households’ nutritional calendar**

1. In which months of the year are food available, no hunger? (*Allow for discussions and record collective answer. Record reasons for the collective answer and opinions of participants)*

| Jan | Feb | Mar | Apr |
| --- | --- | --- | --- |
| May | Jun | Jul | Aug |
| Sep | Oct | Nov | Dec |

2. In which months of the year the foods are likely to be not available, serious food shortage? *Allow for discussions and check all that apply-Record reasons and opinions of participants)*

| Jan | Feb | Mar | Apr |
| --- | --- | --- | --- |
| May | Jun | Jul | Aug |
| Sep | Oct | Nov | Dec |

3. Ask the FGD participants the food their families in general mostly prefer to eat in different periods indicated. (*Allow for discussion and records the common response from the group).*

| S/N. |  | *During the period of food availability* | *During the period of food shortage* | *Remarks (Is there any addition or missing meals between the two periods?)….special observation* |
| --- | --- | --- | --- | --- |
| *1* | *Morning* |  |  |  |
| *2* | *Lunch* |  |  |  |
| *3* | *Dinner* |  |  |  |

*************Asante sana***********
